# Estimating the spawning and growth of striped snakehead *Channa striata* Bloch, 1793 in Lake Rawa Pening Indonesia

**DOI:** 10.1038/s41598-020-76825-5

**Published:** 2020-11-16

**Authors:** Djumanto, Eko Setyobudi, Charles P. H. Simanjuntak, M. Fadjar Rahardjo

**Affiliations:** 1 Laboratory of Aquatic Resource Management, Department of Fisheries, Faculty of Agriculture, Gadjah Mada University, Jalan Flora Gedung A4, Bulaksumur, Yogyakarta, 55281 Indonesia; 2 Department of Aquatic Resources Management, Faculty of Fisheries and Marine Sciences, Bogor Agricultural University, Jl. Agatis Kampus IPB Darmaga, Bogor, 16680 Indonesia

**Keywords:** Behavioural ecology, Freshwater ecology, Behavioural ecology, Biodiversity, Freshwater ecology

## Abstract

The striped snakehead (*Channa striata* Bloch, 1793) is a commercially important fish in Lake Rawa Pening, central Java, Indonesia. This study aims were to investigate their age, growth, and recruitment pattern, through a sampling that was performed monthly, from November 2017 to August 2018. The individual fish was measured for length and weight, and sex was determined. The otoliths were collected, cleaned, and placed on molds to pour epoxy resin. The otoliths were cut to obtain slices and observed under a microscope at 100 × magnification. Subsequently, the age was determined by counting the number of daily rings. It was established that the snakehead spawned every month, with a comparably higher frequency in the new moon than in other phases, and mostly were 3–10 months old, characterized by bigger and older sizes during the rainy season. Furthermore, the average growth rate was observed to be faster during the rainy season in contrast with the dry season. Hence, the parameters of von Bertalanffy growth functions were estimated by back-calculated length, and the pattern in female was Lt = 56.09 (1 − e^− 0.81 (t + 0.07)^), while male Lt = 59.36 (1 − e^− 0.71 (t + 0.09)^), and total Lt = 60.32 (1 − e^− 0.71 (t + 0.14)^), respectively.

## Introduction

The striped snakehead (*Channa striata* Bloch, 1793) is a commercially important freshwater fish, found in a variety of habitats, encompassing swamp, rivers, streams, and a lake in southeast Asia, middle east and Africa^1^. *Channa striata* are locally known as ikan gabus, belonging to the family Channidae^2^, which prey on fish, crustacean, and gastropod^3^. Furthermore, it is one of the promising freshwater species for both semi-intensive and intensive cultures in South East Asia^4^, including Indonesia, with its discovery in Lake Rawa Pening. Also, there is information on their possession of high nutritional and economic value^5^ as well as ecological benefits, with the advantages of being a source of food with very high protein content^6^, and albumin that is very beneficial to health^7^. These are also targeted for fishing, especially in terms of recreation, and ecologically as the top predator, as their existence possibly controls prey populations and species composition^3^.

The demand for snakehead from Lake Rawa Pening is very high and tends to increase, consequently resulting in an upsurge of fresh snakehead meat selling price. This possibly ranges from US4$ to US6$ per kilogram, making it is the main catch target of fishermen, as a large proportion is sent to Semarang and other big cities in Central Java. The fishing process is performed almost throughout the year, using various types of gears, including fishing rods, traps, spears, bamboo slats fence (local name = widik), as well as circular and lift nets. Furthermore, their population in Lake Rawa Pening was observed to have been influenced by some factors, including the number of recruits, growth rate, fishing pressure, and natural deaths due to disease, predation, starvation, or old age. Also, the native stock quantity decreases as a result of intensive fishing methods, use of gears that are not environmentally friendly, and a decline in quality of aquatic biota^8^, which reduce reproductive capacity.

There is high fishing pressure on snakeheads found in the waters of Lake Rawa Pening, and the estimated number caught was about 360 tons/year. This activity directly affects the population size, thus the enhanced tendency to decrease within the last decade. Consequently, the large-scale exploitation causes very high vulnerability, which makes recruitment challenging on instances where the parent stock is deficient, despite that it is below the threshold^9^. Therefore, understanding the biology of snakehead in this habitat is critical, especially in the aspect of growth rate and spawning season, and related research provides the necessary information for achieving sustainable fish management, as well as a general understanding of this fish^10^.

Several studies have previously been carried out by some researchers^11^, its feed evaluation^12^ and domestication^13^ has been investigated. Furthermore, report on reproductive biology in the Musi river flood area was performed^8,14^, while the use of a daily ring of an otolith in determining the age of the snakehead has been conducted in Thailand^15^, however it has never been done in Indonesia. Thus, research on its growth rate and spawning season in Lake Rawa Pening, which is based on daily age, does not exist, making this research fundamental. The aim of this study, therefore, was to examine the growth rate and spawning pattern of snakehead more comprehensively in Lake Rawa Pening, to enable the use of the result as a basis for its resource management.

## Result

### Fish size, age, and hatching date distribution

**Figure 1:**
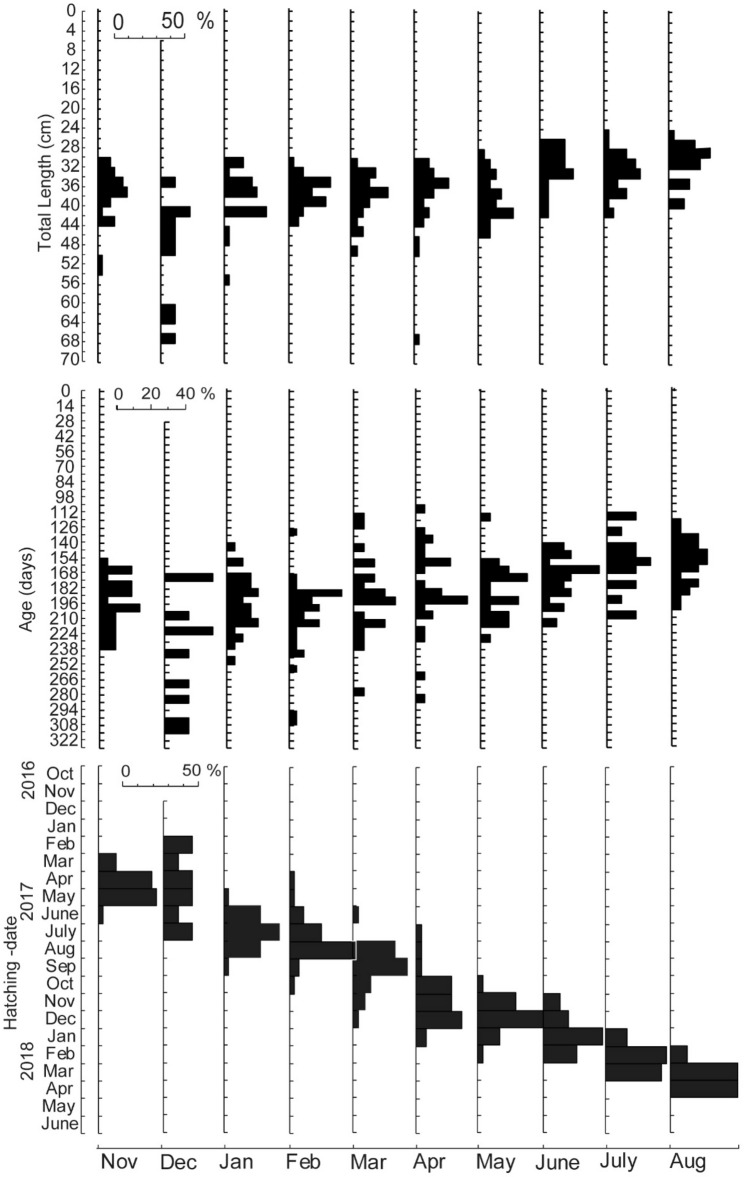
Monthly changes in total length, age and hatching-date distribution of *C. striata* in Lake Rawa Pening, from November 2017 to August 2018.

The number of snakehead otolith samples analyzed was 254 individuals, consisting of 93 females and 161 males, and the length, age and spawning time was presented in Fig. 1.

The fish length was distributed from 23.0 to 65.0 cm, with an average of 33.0 cm, although the monthly measurement majorly spread out at a range of 26.0–46.0 cm. Furthermore, the length distribution in the rainy season (October–March) was comparably wider than in the dry (April-September), at an average of 37.0 cm and 32.0 cm, respectively. These modes became smaller through the sampling month, indicating the occurrence of scheduled recruitment.

Fish age distributed from 100 to 305 days, at an average of 180, although most occurred from 112 to 252 days. This was more extensive in the rainy than the dry season, where the average tends to be younger as seen in the sampling month.

The distribution of hatching-date was throughout the year, from January 2016 to April 2018, and an overlap occurred every month. In addition, the frequency in the dry season was comparably wider than the observation during the rains.

**Figure 2:**
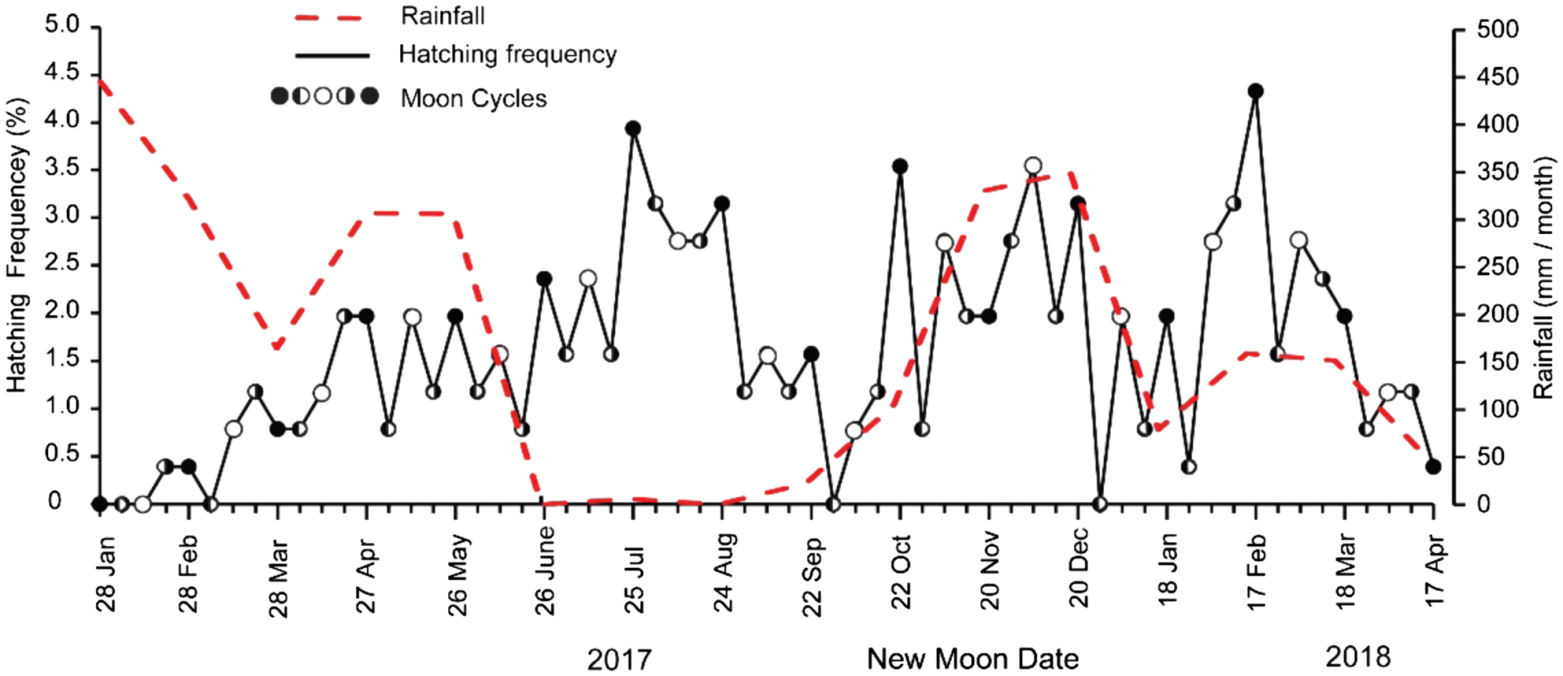
Distribution of hatching-date frequency over the lunar cycle of *C. striata* during 2017–2018, where filled and open circles represent new and full moons, respectively.

The relationship between spawning frequency relative, month cycle, and monthly rainfall are presented in Fig. 2. The rainy season in the study area generally ensues from October to March, although anomalies were recorded in 2017, based on the occurrence of rainfalls in April-June. In addition, the values recorded within the period of 2017–2018 ranged between 40.0–445.0 mm/month, with January exhibiting the highest value^19^. Meanwhile, the spawning frequency relative of snakehead was unaffected by the rainfall recorded in January-June, September-December 2017, and January-April 2018, although it was influenced by the lunar cycle. Therefore, the highest frequency occurred in the new moon (33.46%), followed by the full moon (27.95%), and the third quarter (23.62%), while the lowest ensued in the first quarter (14.96%).

**Figure 3:**
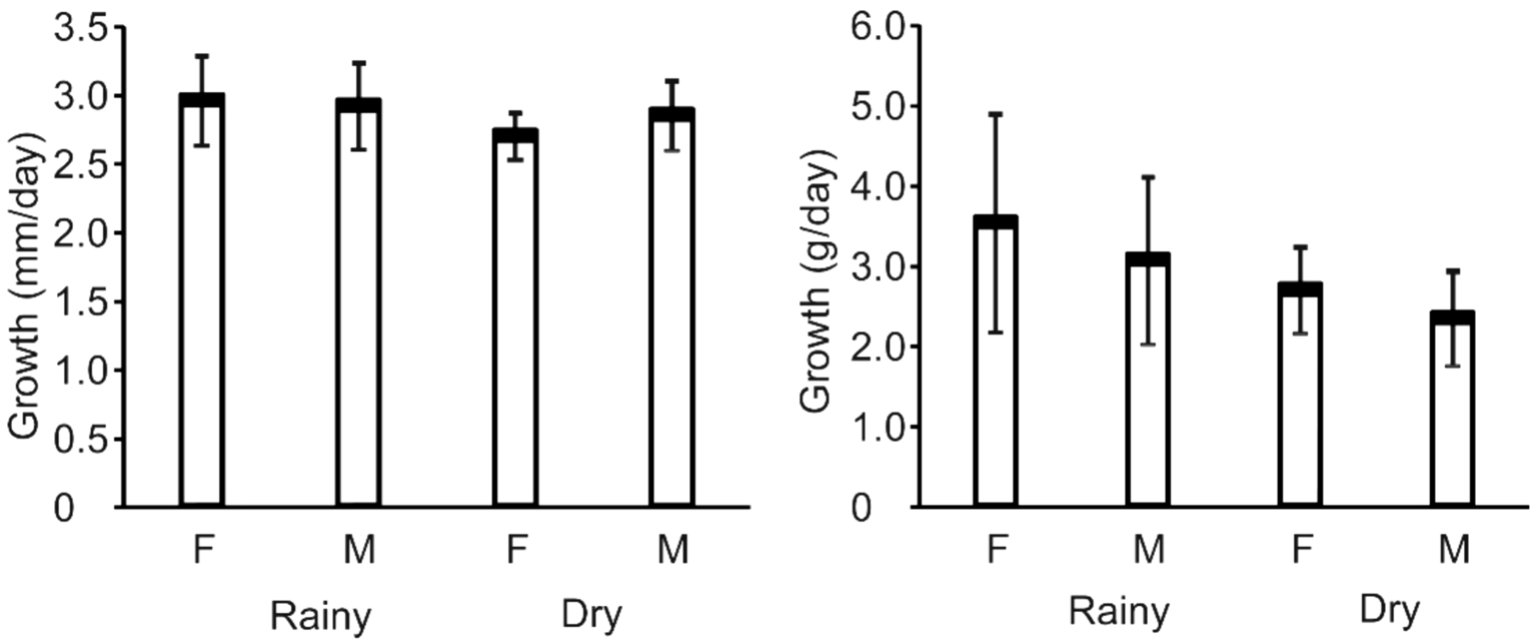
Growth rates of length (mm/day) and weight (g/day) of *C. striata* female and male in the rainy and dry seasons, the bar is a standard deviation.

The growth rate in length and weight of snakehead are presented in Fig. 3. In addition, the rainy season is characterized by a length change of 2.96 ± 0.33 and 2.92 ± 0.32 mm/day for male and female, respectively, while the values in the dry season were 2.70 ± 0.17 and 2.85 ± 0.25 mm/day, respectively. Furthermore, the mean growth rate in terms of weight was 3.53 ± 1.36 and 3.07 ± 1.04 g/day, respectively for female and males in the rainy season, while the values in the dry season were 2.70 ± 0.54 and 2.35 ± 0.59 g/day, respectively. Furthermore, there was no difference in growth rate between both sexes within the seasons, although comparably higher values were recorded during the rains.

**Figure 4:**
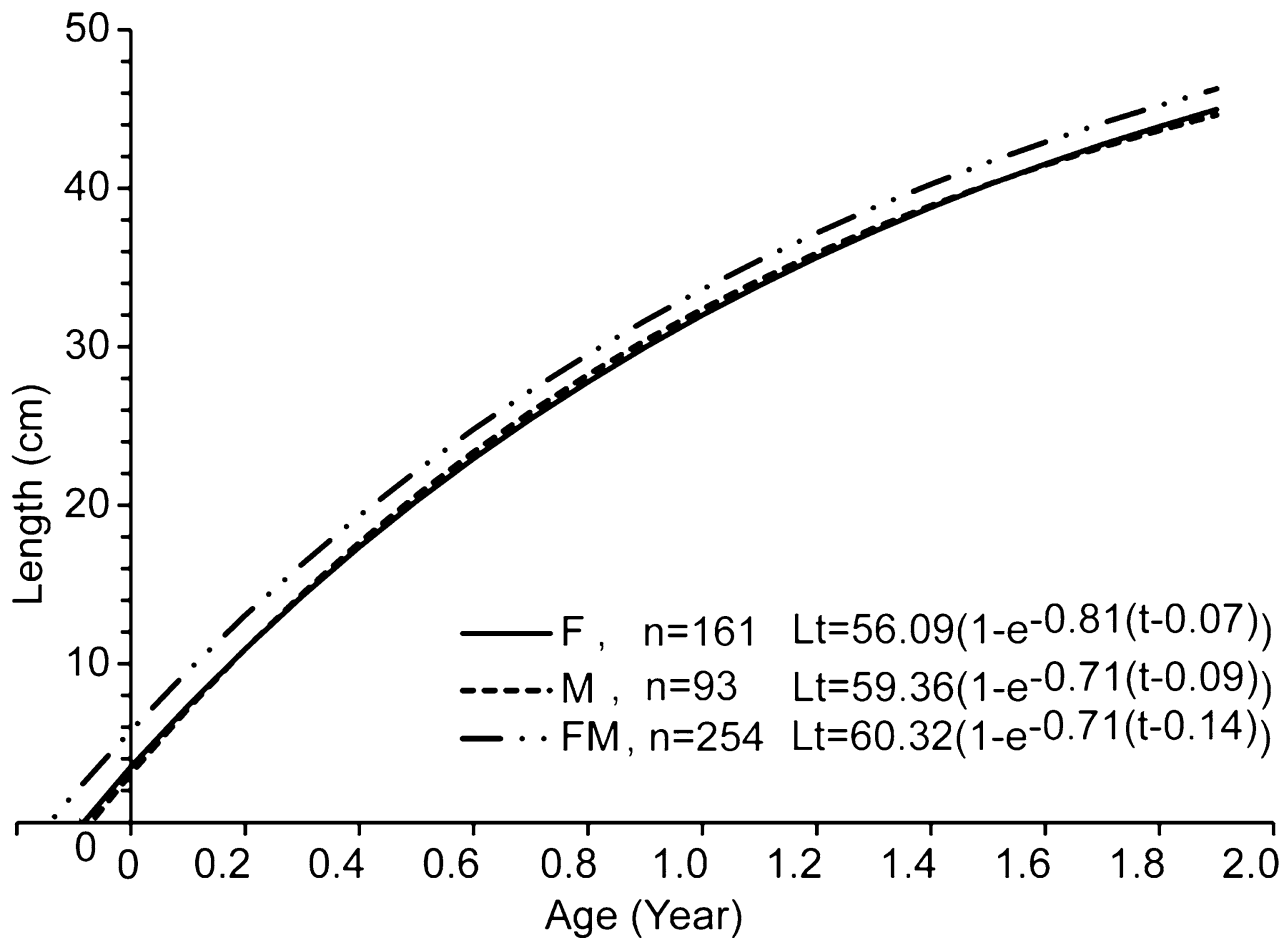
Growth curve patterns of the *C. striata* estimated from the daily ring in otolith, female (solid line) with Lt = 56.09 (1 − e^− 0.81 (t + 0.07)^), male (dash line) was (Lt = 59.36 (1 − e^− 0.71 (t + 0.09)^), while mixed (solid and dash line) was Lt = 60.32 (1 − e^− 0.71 (t + 0.14)^).

The growth function of Von Bertalanffy was adjusted according to the age-length data pair for each sex, and both are presented in Fig. 4. Furthermore, the growth coefficient (k) for female and male were 0.81 and 0.71, respectively, with a collective total of 0.71. The asymptote length was 56.09 cm and 59.36 cm, respectively, and the collective total was 60.32 cm. Meanwhile, at the theoretical age t0, L = 0, which was 0.07 for female, 0.09 for male, and a total of 0.14. Therefore, the von Bertalanffy formula growth curve patterns of the snakehead was Lt = 56.09 (1 − e^− 0.81 (t + 0.07)^) for female, (Lt = 59.36 (1 − e^− 0.71 (t + 0.09)^) for male, and Lt = 60.32 (1 − e^− 0.71 (t + 0.14)^) for mixed, with the female possessing a higher growth coefficient but a shorter length asymptote.

## Discussion

The range of values obtained for snakehead length and weight were wider in contrast with the previous observations^8^, measuring 23.0–65.0 cm in length and 100–1600 g weight. This was possibly due to differences fishing gear used for sampling, which consequently affects the size of the fish caught, and the long duration of sampling^20^. This study involved the use of bamboo blinds as an encircling gear for samples hidden under a grove of water hyacinths and other aquatic weeds^21^. In addition, the snakehead requires water hyacinth as a spawning and nursery ground, which also serves as a hiding place for hunting prey ^13^.

Fish reproduction is influenced by factors that standalone or simultaneously contribute to spawning success, including (1) Internal factors consisting of reproductive hormone concentration, gonadal maturity level, age, and others^9^. (2) External factors, which include water quality, the abundance of food, availability of mates and spawning habitat, as well as the minimized occurrence of predators^20^. In addition, rainfall tends to affect external factors, which include water quantity and quality; hence, most fishes in the tropics generally spawn seasonally alongside the rain cycle, which becomes the main driver^22^.

The island of Java, Indonesia experiences dry season in May–August yearly, characterized by water inundating in river basins, swamps, or lakes, as well as the development or resting stage of gonads, and the absence of spawning. In addition, the arrival of the rainy season in September–April inundates the flood area around rivers, swamps, or lakes, and the high rain causes the movement of many nutrients from the land to the waters, enhancing the fertility of waters, and the abundance of larvae feed. Subsequently, some broodfish tend to migrate longitudinally or laterally, in a search for suitable spawning grounds^22^, for example, whitefish *Barbonymus gonionotus* migrates upstream to obtain fresh water. Meanwhile, blackfish *C. striata* laterally moves to the riverside containing a lot of shrub or grasses^23^, with shallow riverbanks or swamps characterized by numerous varieties of grass, which creates an ideal place for larval growth^9^. Therefore, most fish tend to spawn in the rainy season to ensure an enhanced rate of offspring survival^24^.

Snakeheads spawn during the rainy season in some parts of Asia, e.g., peaking in April at central Laos, which is a period characterized by a rise in water temperature^25^. Conversely, the process takes place in India within June–October^26^, April-July in Bangladesh^27^, and July to November in Taiwan, coinciding with the highest rainfall^28^, and in September-December on the island of Sumatra^14^.

However, snakehead was assumed to spawn monthly at Lake Rawa Pening, indicated by the reduction in average length and age of snakehead sampled each month. Nevertheless, a forward shift in spawning mode is observed following the monthly sampling time, subsequently determining the recruitment period. The mode of length measurement was relatively unchanged, while the hatching-date mode shifts every month. If spawning takes place in a month or time was short, then the hatching-date mode didn't move to indicate a specific month. This characteristic of the collected sample was supposedly due to several factors, encompassing the availability of food for the broodstock and its progeny, and the availability of spawning habitat around the water hyacinth, as well as the relative stability of water quality^9^.

The waters in the sample area were inhabited by 18 species of small fish, dominated by *Osteochillus hasseltii*, and two species of crustaceans, majorly the shrimp termed *Caridina laevis*, which are abundantly available as the primary food for snakehead. Meanwhile, additional feeds include gastropods (*Pomacea canaliculata, Pila ampullacea*), and debris^8^, although the females prefer crustacean (*Caridina laevis, Macrobrachium rosenbergii*) feed, while male prefers fish feed (*Osteochillus hasseltii, Rasbora lateristriata*)^14^. Therefore, the abundant availability of feed is assumed as the reason for the occurrence of the studied species in Lake Rawa Pening for the monthly spawning process.

The primary water source includes surface and underground rivers that supply about 504 million m^3^ per year, while the storage capacity in the dry and rainy season are approximately 25 and 65 million m^3^, respectively. An average discharge rate of 12.3 m^3^/s was recorded from the reservoir, through the Tuntang River^29^; thus, the elevation of Lake Rawa Pening is relatively stable. In addition, the most dominant aquatic weed was *Eichhornia crassipes,* covering 20% of the water surface, with biomass ranging from 15–28 kg of wet weight/m^2^, the density of 40–60 individual plants per m^2^, and plant height of about 50 cm. Also, the overgrowth of aquatic weeds in the study location makes it an excellent habitat for snakehead to breed^21^, alongside the presence of abundant feed and stable water elevation, which actively support spawning throughout the year.

High rainfall can increase the quantity and quality of water in Lake Rawa Pening, but these conditions have not been able to stimulate spawning snakehead. Conversely, spawning is triggered by high rainfall in other freshwater fish, e.g., *Amphilophus labiatus* in Sermo Reservoir^30^, and *Rasbora lateristriata* in Ngrancah River^31^, although the lunar cycle was identified as the stimulant for snakehead. Furthermore, the highest frequency was recorded in the new moon, implicating moonlight in the synchronization of reproduction activities. Also, most snakeheads tend to spawn during this period to minimize the predation of their eggs, and they are also nocturnal animals with very sharp eyesight that is relevant in warding off progeny predators. This high spawning frequency in the new moon also occurs in the Mummichog, *Fundulus heteroclitus*^32^, which is in contrast with *Sparus aurata* that occurs in full moon^33^.

Daily growth rates in length and weight were comparably higher in the rainy than the dry season, assumed to occur because the water entering the reservoir contains numerous nutrients needed for the growth of plankton and biotic organisms. In addition, the inflow to the lake emerges from the agricultural area; hence, the fertilizer parts containing dissolved phosphorus and nitrogen are carried by the current into the reservoir. This leads to the seasonal fluctuation in plankton abundance and macroinvertebrate biomass, which was comparably higher in the rainy season^29^.

The growth rate of females was observed to tend to be higher than males, and one of the influencing factors was the feed type and activity level. In addition, the females tend to prey more on crustaceans with higher protein content, than on fish, which was the preference for males, subsequently creating greater fulfillment^34^. Also, the males hunt for prey, while females dwell under the hordes of water hyacinth. Meanwhile the daily growth rate in the aspect of length and weight for males and females in Lake Rawa Pening was relatively higher than the values obtained for those cultivated in hapa nets, which possess an average initial weight and length of 6.6 g and 7.7 cm, respectively for seven months, and a resulting final value of 160 g and 20.8 cm. Therefore, the daily cultivation growth rate was 0.76 g/day and 0.62 mm long^35^, where the differences were presumably due to the abundant availability of snakehead feed on Lake Rawa Pening in the form of diverse species. This turned out to capably meet the nutritional needs and subsequently enhance the speed of growth.

Based on the parameters of growth rate and asymptote length, the samples displayed high indices, as fish with large k have a short life, and the inverse is assumed for those with small k^36,37^. Therefore, the asymptote length for females and males was 56.09 cm, and 59.36 cm, respectively, with a total of 60.32 cm, indicating the small length nature of snakehead. In addition, fish with small total length and a large k value tend to be short-lived because of a limitation in the time required for them to reach maximum length, which was the characteristics of samples in the study location, hence their brief lifetime (< 5 years)^35^. In addition, higher growth is assumed to occur as a result of the abundant food choices to be preyed on, encompassing fish, crustacean as the main menu, while shellfish, insects and detritus served as supplements^28^.

Snakeheads in Lake Rawa Pening exhibit a remarkably higher growth parameter, in contrast with those sourced in Lubuk Lampam Floodplains, South Sumatera province, Indonesia k = 0.36^38^, and in Sri Lanka k = 0.35–0.40^39^. This indicates the fertility of the study location in the aspects of providing enough food for nekton, especially snakehead, being a top predator in the ecosystem. They have been known to prey on several species’ groups of fish, crustaceans, mollusks, annelids, and insects, with preference to small fish and shrimp^4^. However, some often identified prey fish include *Trichogaster pectoralis, Trichogaster trichopterus, Osteochillus hasseltii, Oreochromis mossambicus*, and other species with body length < 0.5 of the snakehead^13^. These varieties tend to grow abundantly, in order to meet the food or nutritional requirements, and the addition of a suitable habitat collectively triggers the high growth of snakehead^37,40^.

The growth rate of snakehead in Lake Rawa Pening, compared to the growth rate in the culture system, was relatively higher. Snakehead growth in aquarium rearing with the feeding of bloodworms reached 0.12 cm/day^41^, while the average snakehead growth rate in the Lake Rawa Pening reached 0.18 cm/day. The higher snakehead growth rate in Lake Rawa Pening is thought to be caused by several factors. Firstly, abundant and varied feeds were available so that the nutrients were enough. In contrast, the snakehead cultivated in aquarium feeding bloodworms only caused nutrients lacking. Secondly, snakeheads that live in a lake have a vaster space so that the metabolism was higher, while cultivated in the aquarium causes limited movement. Thirdly, in the age group 1 year, growth estimation using length-frequency data will result in lower k values than using otolith data^42^.

Their development in the study location was influenced by internal factors, including genetics, health, stage of life, metabolic rate, and external influences, which consist of prey types and availability, as well as competition^19,43^. Within the growing phase, the young snakehead tends to display a high metabolic rate, characterized by an elevation in appetite^4^, and a large portion of net energy metabolism is set aside for vegetative growth^33^. The abundance of water hyacinth and other aquatic plants is identified as an ideal habitat for hiding and searching for snakehead, subsequently minimizing the energy expended in hunting prey. These conditions were, therefore, concluded to be the major growth triggers in Lake Rawa Pening.

## Methods

### Sampling station

**Figure 5:**
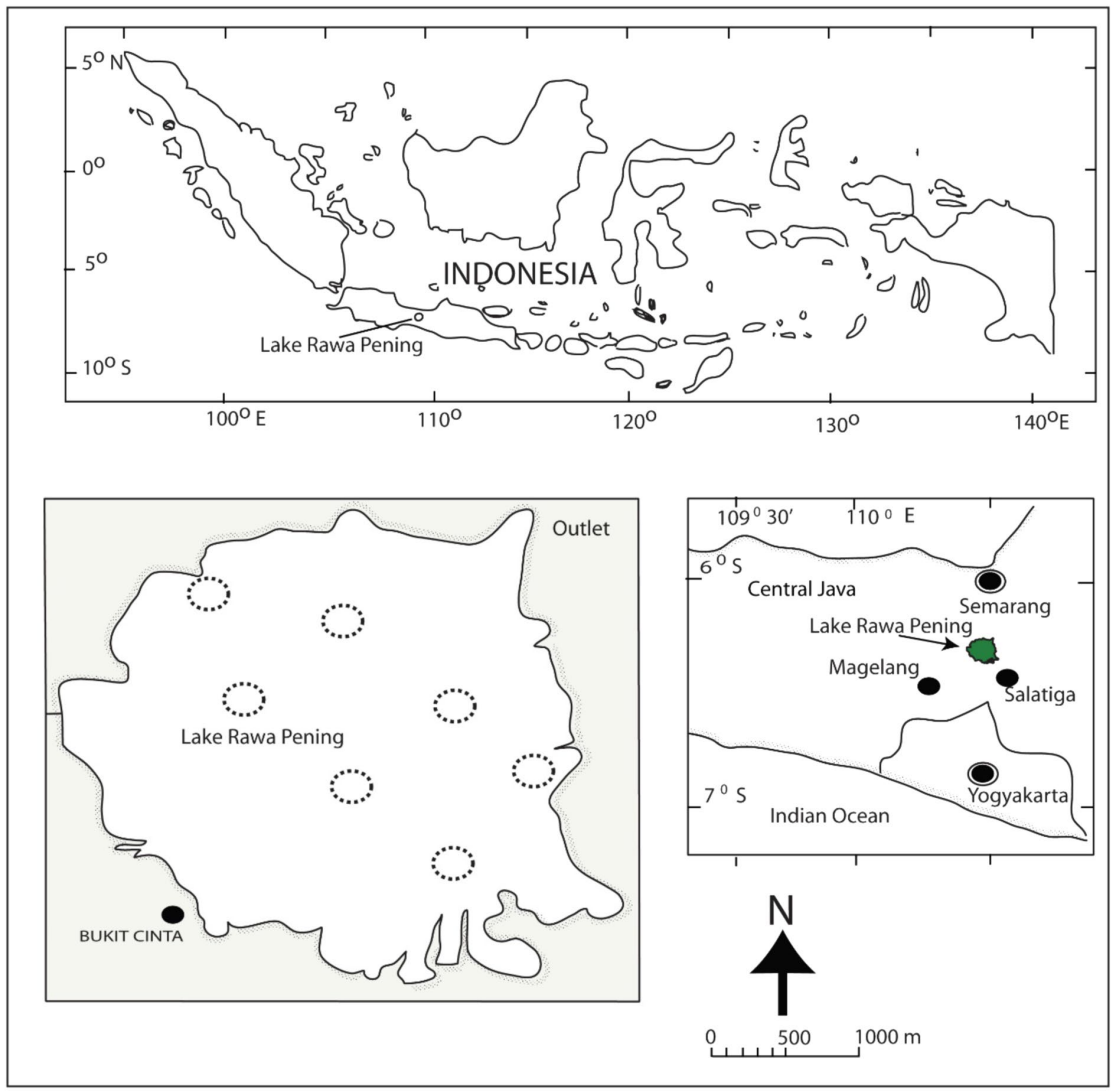
The map shows the location of Lake Rawa Pening in Central Java, as a place for *C. striata* sampling, with stations indicated by a broken circle line. This area was surrounded by hills in the south, west, and north, while the east was a characteristic lowland rice field. In addition, it is oval dash, with inlet channels from the river around the lake to the south, west, and north, while the outlet leads northeast.

Lake Rawa Pening has a maximum area of about 2670 ha and is in Indonesia's Central Java Province. The water source emerges from several rivers, which empties into the west and south sides, and the morphometric of the elliptical lake extends towards the outlet is located northeast. Consequently, seasons of the year affect water level and surface area of the lake, as an area of about 2670 and 1650 ha, with an average depth of 2.5 and 1.5 m recorded in the rains and dry season, respectively. In addition, aquatic weeds tend to cover approximately one-fifth of the surface area, especially in shallow waters on the west and south sides, where water hyacinth (*Eichhornia crassipes*) emerges as the most dominant, with the highest density on the west side of the lake, and the recorded annual DO (dissolved oxygen) concentrations were above 3 mg/L. Furthermore, snakehead sampling was performed in Lake Rawa Pening (Fig. 5), within a monthly interval, from November 2017 to August 2018, which covers the dry and rainy season. Fishing activities were carried out in several locations, representing Bawen and Ambarawa districts, while sampling was performed in areas with a higher density of water hyacinth, which serves as a suitable habitat.

### Fish sampling

The method of catching fish samples conducted in this study was under the guidelines and regulations of the Republic Indonesia government, and the fishing practice was carried out from generation to generation by local fishermen. The research material was snakehead obtained from Lake Rawa Pening. Snakehead hiding under the water hyacinth bunch were caged using bamboo blinds. These fences were made of several sheets of woven bamboo blades measuring 1.5 × 15 m^2^, with a gap of 1 cm, joined into one long sheet of screens. Local fishers assisted the operation of bamboo blinds during sampling. Collectively, they were enclosed in the water hyacinth bunch, and the water hyacinth was removed from the fences during the process of cage size reduction, and the trapped fish were collected using a scoop net. Furthermore, fishing was conducted in the morning up to the afternoon. All captured snakeheads were put into an icebox to maintain freshness, which was then transferred to the laboratory for further observation and analysis.

The width of the barrier blinds possibly influences the size of fish caught, as juvenile stages tend to escape through the gaps, while the young and adults are perhaps trapped in cages; thus, the placement was in a way that optimizes fish collection. In addition, the shape of snakehead is depressed on the head and rounded on the body parts, further increasing the chance of being trapped; hence it is assumed that the samples collected represent the population in Lake Rawa Pening.

In the laboratory, measurements were taken for total length to the nearest mm (L, 0.1 mm), individual weight, using an electric scale to the lowest gram (W, 0.1 g), then their stomach was dissected, and gonads removed for observation and sex determination. This inspection was based on gonadal or testicular ownership, while signs including color, size, texture, and shape of the gonads were evaluated in fish with prematurely developed gonads^16^.

### Otolith preparation

All experimental protocols in this study followed government regulations, and the fish studied were collected from the catches of local fishers so that they did not require ethical clearance. Sagitta was the otolith used for daily ring analysis, located in the sacculus chamber, and its removal of the otolith involves cutting the jawbone, and widely opening the oral cavity. Also, muscle the palate was cut and opened, as well as the head cavity, to obtain clear visualization. Therefore, both otolith sagitta were taken out with forceps, washed, and cleaned with tap water, followed by dry aeration and storage in vial plastic bottles.

Plastic containers measuring 2 × 5 × 2 cm^3^ were used as molds to which 5 mm depth epoxy resin were poured and allowed to harden. Therefore, the sagittal otoliths were marked with flat lines on the distal sides, using a pencil as a cutting point, where the horizontal dash follows the center, and labels were installed on each container to prevent errors. In addition, the samples were placed in a mold, and epoxy resin was poured to attain full immersion of the surface area, and then allowed to harden, at a thickness of about 10 mm.

**Figure 6:**
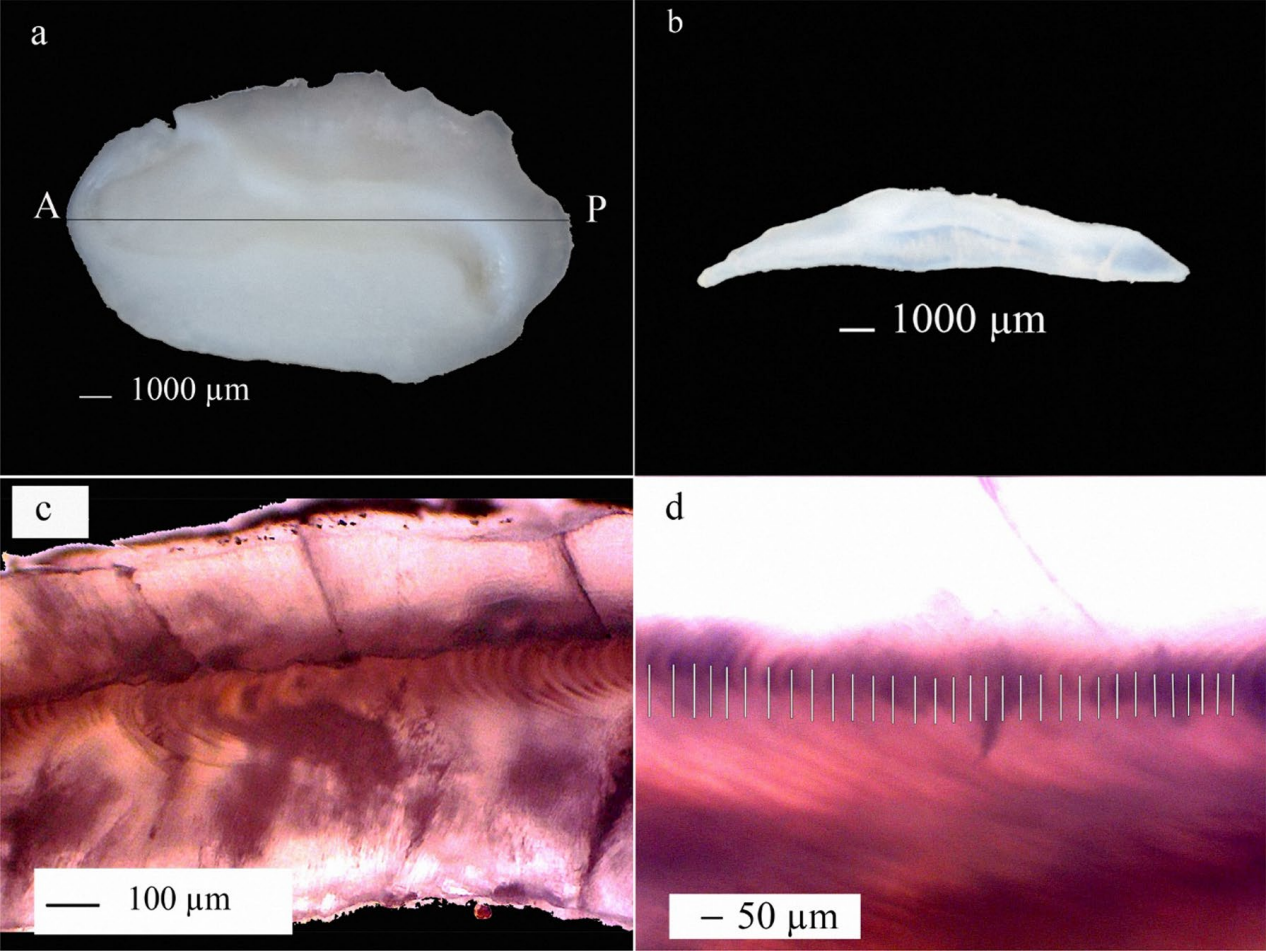
Plate **(a)** photograph showing the dorsal view sagittal otolith of *Chana striata*, 180-days-old male, and a total length (TL) of 370 mm. Plate **(b)** the cut otolith was stretched longitudinally in the anterior (A)–posterior (P) end direction, resulting in a thin slice of 0.3–0.4 mm in thickness. Plate **(c)** Clearly visible daily ring along the middle of the otolith slice, and Plate **(d)** daily ring calculation from the core to the outermost ring.

Otoliths were sliced with a pair of circular ceramic blades, at speed set at 2000–2500 rpm, following the marked line that encompasses the anterior–posterior end longitudinal direction in its center. Beneath the blade lays a water box to soak the knife, which minimizes dust and knock that emerge from the otolith and molds. Therefore, the thickness of a slice maintained between 0.3–0.4 mm was cleaned, placed on an objective glass preparation, subsequently glued with clear nail polish and allowed to dry. A sample was further observed in a light microscope, at 100–200 times magnification, to examine the daily ring, and the right otolith was used for daily ring analysis, although the left otolith was also cut in a similar way if damage ensues in the cutting process (Fig. 6).

The daily range was calculated as the number of translucent zones identified on a standard axis, along the sulcus. Furthermore, each otolith was read by the same reader three times, from the core to the anterior edge, and then averages were calculated to estimate age^15^. The physical data were not provided to the reader to maintain unbiased reading. The otoliths used for analysis are visible daily rings and the difference between readings < 5%.

### Data analysis

The age was calculated as the number of daily rings in each otolith, following the reception of exogenous feeding. Therefore, birthdays were estimated based on the difference between the number obtained and the date of catch.

The hatching-date analysis was performed by back-calculation of the difference between fishing date and age. The examination was carried out using the data processing program, the "exel" with the transformation of dates and numbers. Initially, the fish capture date was transformed into a number subsequently subtracted by the individual's age, and a hatching number was obtained. Then the hatching number was converted back to the date as the hatching-date.

The spawning frequency was calculated based on the number of hatches per all hatches of the lunar cycle, in percent. In one lunar cycle, there were four lunar phases, namely new moon, first quarter, full moon, and the third quarter. The hatch date of all samples was grouped into the lunar phase so that spawning frequency was obtained in each lunar phase.

The daily growth rate, in terms of length and weight, was calculated using the following formula. Daily growth rate in length (mm/day) = total length (mm)/age (day). Daily growth rate in weight (g/day) = weight (g)/age (day).

**Figure 7:**
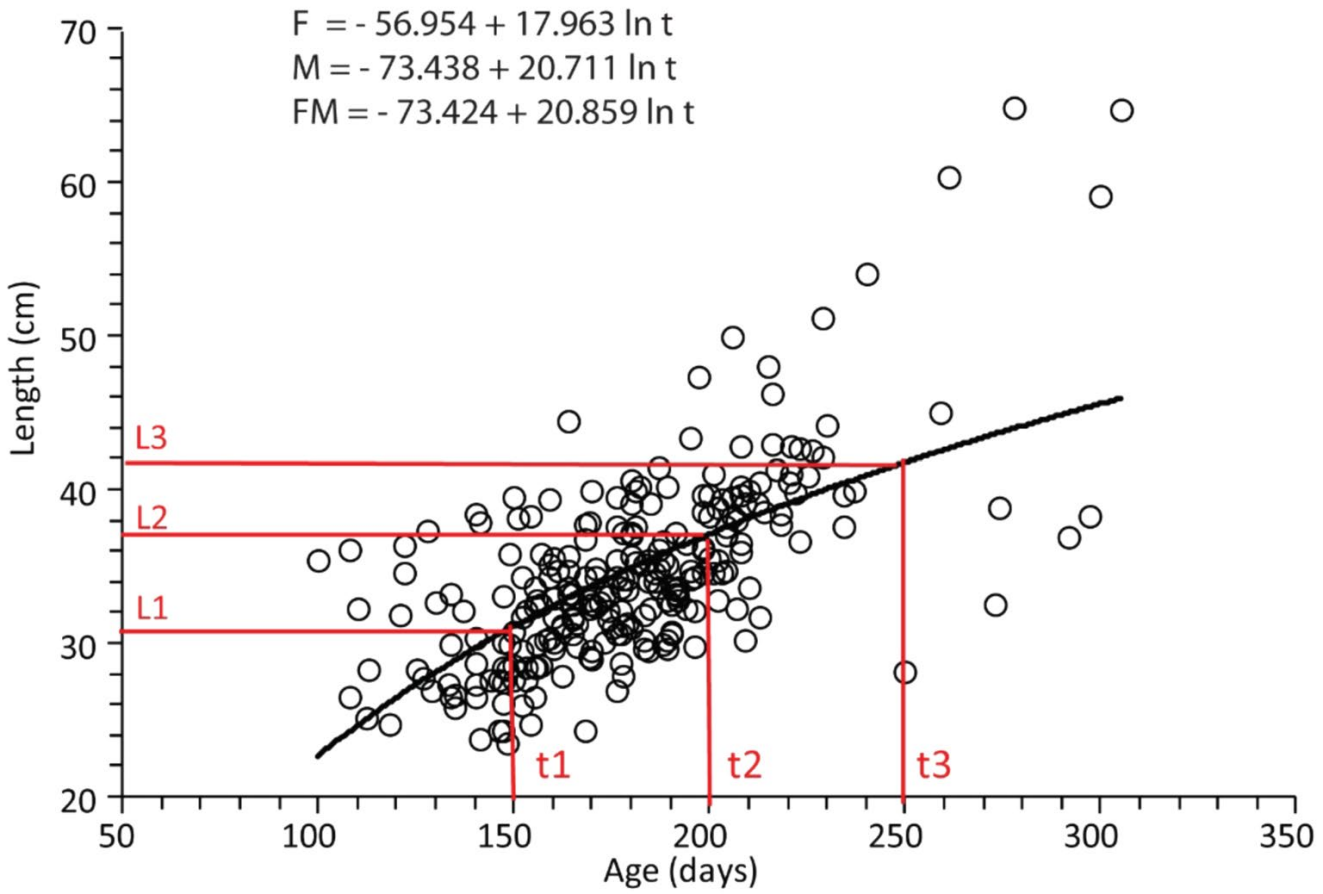
The illustration follows the Francis procedure in determining the values of L1, L2, L3, traced from t1, t2, t3, respectively, using logarithmic equations. The top panel presents the logarithmic equation of male, female, and a combination of both was used to determine the L1, L2, L3 traced from t1, t2, t3, respectively.

The von Bertalanffy growth formula parameter was computed^17^, and the relationship between length and the daily ring involved a non-linear equation, hence obtained at a certain age, as shown in Fig. 7.

Furthermore, the equation showed the estimated length of l_1_, l_2_, l_3_ at the age of t_1_, t_2_, t_3_, respectively, and the points were used to determine the ratio value for growth speed at older to younger age stages as follow.

$$\mathrm{r}=\frac{{\mathrm{l}}_{3}-{\mathrm{l}}_{2}}{{\mathrm{l}}_{2}-{\mathrm{l}}_{1}}$$

where l_1_, l_2_, and l_3 _are the mean lengths at ages t_1_, t_2_, and t_3_, respectively.

The von Bertalanffy parameters include k, L∞, and t_0_ were estimated by the following formula.

$${\mathrm{l}}_{\infty }={\mathrm{l}}_{1}+\frac{{\mathrm{l}}_{3}-{\mathrm{l}}_{1}}{1-({\mathrm{r}}^{2})}$$

$$\mathrm{k}=\frac{-2\mathrm{log}(\mathrm{r})}{{\mathrm{t}}_{3}-{\mathrm{t}}_{1}}$$

$${\mathrm{t}}_{0}{=\mathrm{t}}_{1}+\frac{1}{\mathrm{k}}\mathrm{log}(\frac{{\mathrm{l}}_{\infty }-{\mathrm{l}}_{1}}{{\mathrm{l}}_{\infty }})$$

where Lt is the standard length at age t, L∞ is the asymptotic standard length, k is the growth coefficient, t is the age, and t0 is the theoretical age at zero length^18^.

## References

[1] Beamish, F.W., Beamish, R.B. & Lim, S.L. Fish assemblages and habitat in a Malaysian blackwater peat swamp. *Environ. Biol. Fish.* **68**, 1–13, 10.1023/A:1026004315978 (2003)

[2] Nelson, J.S., Grande, T.C.& Wilson, M.H. *Fishes of the World*. (Wiley, 2016)

[3] Nurdawati N, Husnah A, Prianto E (2007). Fish fauna in peat swamp lake in South Barito, Central Kalimantan. J. Iktiol. Indo..

[4] Belinda MSW, Beamish FWH (2004). Ontogenetic changes in morphology and diet in the snakehead, *Channa limbata*, a predatory fish in western Thailand. Environ. Biol. Fish..

[5] Suwandi R, Nurjanah A, Winem M (2014). Proportion of body parts and proximate content of snakehead at various sizes. J. Indo. Fish. Prod. Proc..

[6] Haniffa MA, Nagarajan M, Marimuthu K, Jesu AAR (2003). Embryonic and larval development of spotted murrel, *Channa punctatus* (Bloch). J. Indian Fish..

[7] Romadhoni AR, Afrianto E, Pratama RI, Grandiosa R (2016). Extraction of snakehead fish [*Ophiocephalus striatus* (Bloch, 1793)] into fish protein concentrate as albumin source using various solvent. Aqua. Proc..

[8] Puspaningdiah M, Solichin A, Ghofar A (2014). Biological aspects of snakehead (*Ophiocephalus striatus*) in the waters of Rawa Pening lake, Semarang Regency. J. Maq. Dip..

[9] Amilhat E, Lorenzen K (2005). Habitat use, migration pattern and population dynamics of chevron snakehead *Channa striata* in a rainfed rice farming landscape. J. Fish Biol..

[10] Gao Y, Feng Q, Ren D, Qiao L, Li S (2010). The relationship between trace elements in fish otoliths of wild carp and hydrochemical conditions. Fish Physiol. Biochem..

[11] Muntaziana NA, Rahman AM, Rahim AA, Marimuthu K (2013). Present culture status of the endangered snakehead, *Channa striatus* (Bloch, 1793). J. Anim. Vet. Adv. Asia..

[12] Zehra S, Khan MA (2011). Dietary protein requirement for fingerling *Channa punctatus* (Bloch), based on growth, feed conversion, protein retention and biochemical composition. Aquacult. Int..

[13] Ndobe S, Serdiati N, Moore A (2014). Domestication and length-weight relationship of striped snakehead *Channa striata* (Bloch).

[14] Makmur S, Rahardio MF, Sukimin S (2003). Biology of reproduction of snakehead (*Channa striata* Bloch) in the Musi river flood area of South Sumatra. J. Iktiol. Indo..

[15] Jutagate,T., Phomikong,P., Avakul, P. & Saowakoon, S. Age and growth determinations of chevron snakehead *Channa striata* by otolith reading. in: *Proceedings of the 51st Kasetsart University Annual Conference, Bangkok, Thailand, 5–7 February 2013, 11–17* (2013)

[16] Ghaedi A, Kabir MAA, Hashim R (2013). Oocyte development and fecundity of snakehead murrel, *Channa striatus* (Bloch 1793) in Captivity. Asia. Fish. Sci..

[17] Francis RICC (1988). Are growth parameters estimated from tagging and age length data comparable?. Can. J. Fish. Aqua. Sci..

[18] von Bertalanffy L (1938). A quantitative theory of organic growth (inquiries on growth laws II). Hum Biol..

[19] Anonim. *Climatology Station of Lake Rawa Pening. BPSDA Central Java Prov*. https://bpusdataru-bk.jatengprov.go.id/index.php/informasi-sda/hidrologi/klimatologi/pos-klimatologi-rawa-pening (2018).

[20] Payan P, Borelli G, Boeuf G, Mayer-Gostan N (1998). Relationship between otolith and somatic growth: Consequence of starvation on acid-base balance in plasma and endolymph in the rainbow trout *Oncorhynchus mykiss*. Fish Physiol Biochem.

[21] Ali AB (1999). Aspects of the reproductive biology of female snakehead (*Channa Striata*bloch) obtained from irrigated rice agroecosystem, Malaysia. Hydrobiology.

[22] Chellappa S, Bueno RMX, Chellappa T, Chellappa NT, Almeida e Val VMF (2019). Reproductive seasonality of the fish fauna and limnoecology of semi-arid Brazilian reservoirs. Limnology.

[23] Tongnunui S, Beamish FWH (2009). Habitat and relative abundance of fishes in small rivers in eastern Thailand. Environ. Biol. Fish..

[24] Breeding S-I, Growth L (2017). Yulintine, Bugar, H., Wulandari, L., Harteman, E. Snakehead Fish (Channa Striata). J. India. Sci. Tech..

[25] Morioka S, Vongvichith B, Chanthasone P, Phommachane P, Suzuki N (2016). Reproductive season, age estimation and growth in a striped snakehead Channa striata population in Nasaythong District, Vientiane Province, Central Laos. Aquacult. Sci..

[26] Prasad L, Dwivedi AK, Dubey VK, Serajuddin M (2011). Reproductive biology of freshwater murrel, *Channa punctatus* (Bloch, 1793) from river Varuna (A tributary of Ganga River) in *India*. J. Ecophysiol. Occup. Health..

[27] Ferdausi HJ, Roy NC, Ferdous MJ, Hossain MA, Hasan MM (2015). Reproductive biology of striped snakehead (*Channa striata*) from natural wetlands of Sylhet, Bangladesh. Ann. Vet. Anim. Sci..

[28] Li K, Shieh B, Chiu Y, Huang D, Liang S (2016). Growth, diet composition and reproductive biology of the invasive freshwater fish chevron snakehead *Channa striata* on a subtropical island. Zool. Stud..

[29] Goeltenboth F, Kristyanto AIA (1994). Fisheries in the Rawa Pening Reservoir, Java, Indonesia. Int. Rev. Hydrobiol..

[30] Habibie SA, Djumanto, Rustadi A (2015). The use of otolith to determine age and spawning time of red devil (*Amphilophus labiatus* Günther, 1864) in Sermo Reservoir, Yogyakarta. J. Iktiol. Indo..

[31] Sentosa AA, Djumanto (2010). Spawning habitat of *Rasbora lateristriata* in Ngrancah River, Kulon Progo Regency. J. Iktiol. Indo..

[32] Taylor MH, Leach GJ, Dimichele L, Levitan WM, Jacob WF (1979). Lunar spawning cycle in the mummichog, *Fundulus heteroclitus* (Pisces: Cyprinodontidae). Copeia.

[33] Saavedra M, Ferreira PP (2006). A preliminary study on the effect of lunar cycles on the spawning behaviour of the gilt-head sea bream, *Sparus aurata*. J. Mar. Biol. Ass. UK.

[34] Mohanty SS, Khuntia BK, Sahu B, Patra S, Tripathy K (2017). Effect of feeding rates on growth, feed utilisation and nutrient absorption of murrel fingerling, *Channa striata* (Bloch) and determination of protein and energy requirement for maintenance and maximum growth. J. Nutr. Food Sci..

[35] Anonim. *Academic Subject of Snakehead (Channa striata bloch 1793) Results of Domestication*. (Mandiangin Freshwater Fisheries Research, Directorate General of Fisheries Aquaculture, Ministry of Marine and Fisheries, 2014) **(in Indonesia)**.

[36] Sparre, P. & Venema, S.C. Introduction to tropical fish stock assessment. in: *Part 1: Manual. FAO Fisheries Technical Paper No. 306 /1 Rev.2* (1998).

[37] Garrido S, Ben-Hamadou R, Santos AMP, Ferreira S, Teodósio MA, Cotano U, Irigoien X, Peck MA, Saiz E, Ré P (2015). Born small, die young: Intrinsic, size-selective mortality in marine larval fish. Sci. Rep..

[38] Fahmi Z, Nurdawati S, Supriyadi F (2013). Growth and exploitation status (*Channa striata* bloch, 1793) in lubuk lampam floodplains, South Sumatera. J. Fish. Res. Indo..

[39] De Silva KHGM (1994). The fishery, growth rates and notes on reproduction of the snakehead *Channa striata* (Bloch) (Perciformes: Channidae) in some irrigation reservoirs of Sri Lanka. Fish. Res..

[40] Palstra, A.P. & van den Thillart, G.E.E.J.M. Swimming physiology of European silver eels (*Anguilla anguilla* L.): energetic costs and effects on sexual maturation and reproduction. *Fish Physiol. Biochem.* **36**, 297–322, 10.1007/s10695-010-9397-4 (2010). 10.1007/s10695-010-9397-4 PMC2923712 20390348

[41] Saputra A, Budiardi T, Samsudin R, Rahmadya ND (2018). Growth performance and survival of snakehead *Channa striata* juvenile with different stocking density reared in recirculation system. J. Akuakultur Indonesia..

[42] Hammers BE, Miranda LE (1991). Comparison of methods for estimating age, growth, and related population characteristics of white crappies. N. Am. J. Fish. Manag..

[43] Arai, T. & Chino N. Opportunistic migration and habitat use of the giant mottled eel *Anguilla marmorata* (Teleostei: Elopomorpha). *Sci. Rep.* **8**:5666, 10.1038/s41598-018-24011-z (2018). 10.1038/s41598-018-24011-z PMC5884785 29618838

